# Immunoglobulin G4 deficiency can be a new entity for primary recurrent miscarriage: Successful pregnancy in two cases after treatment with intravenous immunoglobulin

**DOI:** 10.4274/tjod.galenos.2019.02650

**Published:** 2020-02-28

**Authors:** Behzad Shakerian, Mozhgan Moghtaderi, Nasrin Heidari

**Affiliations:** 1 Shariati Hospital, Clinic of Immunology, Isfahan, Iran; 2 Shiraz University of Medical Sciences, Neonatal Research Center, Shiraz, Iran; 3 Fertility and Infertility Center, Isfahan, Iran

**Keywords:** Recurrent miscarriage, intravenous immunoglobulin, immunoglobulin G, female infertility

## Abstract

Recurrent miscarriage is one of the complications of pregnancy in which the potential role of immunologic factors has already been mentioned. Here, two young women with recurrent miscarriage were consulted in the infertility center. The diagnosis of immunoglobulin G4 (IgG4) deficiency was made through the reduction of IgG4 Ig levels and normal total IgG titer. Considering this abnormality, intravenous Ig 200 mg/kg was started monthly, and they both had successful pregnancies. Little is known about IgG4 deficiency in women with recurrent miscarriage. IgG4 deficiency should be taken into account in these patients. It is expected that these results will shed further light on the feasibility of intravenous Ig for women with recurrent miscarriage.

**PRECIS:** Heterotopic pregnancy treated with vNOTES procedure

## Introduction

Recurrent miscarriage (RM) is considered as ≥2 fetal losses before 24 weeks of gestation, affecting an estimated 1-3% of women in their reproductive age. It is classified as primary RM without a previous viable pregnancy and secondary RM with one or more pregnancies^(1)^. The etiology of RM in up to 50% of cases is genetic. Thyroid disorders have a disputed role in RM, and investigations for thyroid-stimulating hormone and thyroid antibodies are strongly recommended in RM. Searching for uterine malformations should also be considered as a part of diagnostic examinations of couples with RM. There is a doubtful association between hereditary thrombophilia and acquired thrombophilia or Antiphospholipid syndrome with miscarriage. Antiphospholipid syndrome is diagnosed through the persistent presence of antiphospholipid antibodies and vascular thrombosis; the role of alloimmune mechanisms remains poorly understood^(1,2,3)^. The role of immunologic factors has already been mentioned for reproductive failure in patients with RM. A normal pregnancy induces some changes in antibody formation; increased level of immunoglobulin M (IgM) at the early phase of a primary immune response and IgE antibodies in some mothers with a genetic background. At the late phase of primary immune response or after chronic antigen exposure, antibodies switch to IgG; IgG4 in particular have received special attention^(4,5)^.

IgG4 accounts for approximately 5% of total IgG in the serum of adults and is unable to activate the classic complement pathway and antibody-dependent cell-mediated cytotoxicity. IgG4 uniquely plays as a blocking antibody with anti-inflammatory properties; therefore, it is known a as a rule-breaker antibody^(6,7)^. IgG4 deficiency is the most common immunodeficiency subclass. A deficiency can be identified as a level >2 SD less than age-matched reference values accompanied with a normal total IgG level^(8)^. Some patients with decreased IgG4 are asymptomatic, but several groups present with recurrent respiratory infections. IgG4 deficiency has been noted in some primary immunodeficiency disorders such as Wiskott-Aldrich syndrome, IgM deficiency, ataxia telangiectasia, and mucocutaneous candidiasis. Other disorders with IgG4 deficiency are Down syndrome, immune thrombocytopenia, lupus erythematosus, and growth hormone deficiency. Monthly intravenous immunoglobulins (IVIG) are the mainstay of treatment for symptomatic patients with IgG subclass deficiencies^(9,10)^. There are scant data on abnormal Ig subclasses in women with RM^(11)^. Due to pregnancy loss and its repetitive nature in women, and even its emotional impact on men, there is a need for studies on the etiologic factors of RM. Little is known about IgG4 deficiency in women with RM. We report two young women with primary RM and IgG4 deficiency who became pregnant after IVIG therapy.

## Case Reports

### Case 1

A 24-year-old Caucasian woman, gravida 2 parity 0, with a history of two miscarriages was seen in Isfahan Fertility and Infertility Center affiliated to Isfahan University after five years of marriage. The ages of miscarriage were 8 weeks and 10 weeks’ gestation. She was not a smoker, she had no significant past medical history and was not on any long-term medications. Her physical examination was normal with body mass index (BMI) of 26.6 kg/m^2^. Her menstrual cycles were regular (every 28-30 days with 6 days of menstrual flow). The results of hormonal investigations revealed normal gonadotropin levels, implying regular ovulatory cycles. Transvaginal ultrasound of the pelvis showed a normal uterus and ovaries. Based on her history and laboratory features, a diagnosis of unexplained RM was made. Evaluation of blood parameters revealed white blood cells (WBC) 8200 cell/mm^3^, hemoglobin (Hb) 14.3 g/dL, and platelets 264000/mm^3^. The laboratory tests for diabetes, hyperprolactinemia, and vitamin D deficiency were normal. Antinuclear antibody and anti-dsDNA levels were measured using enzyme-linked immunosorbent assay with a negative result. The other antibody identification studies are shown in Table 1. Considering the history of RM with no causes, further immunologic investigations were performed (Table 2). The detection of low serum IgG4 levels was significant. Treatment with IVIG (Biotest’s Intratect^®^, Germany) was started with 200 mg/kg monthly with the diagnosis of antibody deficiency; she became pregnant after two months. Subsequently, the woman had a successful pregnancy and her infant was delivered at 37 weeks of gestation with a normal Apgar score.

### Case 2

A 27-year-old woman with a history of eight miscarriages in the second trimester was referred from Isfahan Fertility and Infertility Center affiliated to Isfahan University. Her age at marriage was 17 years. She had regular menstrual cycles since the age of 13 years. There was no history of smoking and other diseases. At the time of her referral, her BMI was 26.8 kg/m^2^. Transvaginal ultrasound revealed no anomalies of the uterus and ovaries. The initial complete blood count revealed WBCs of 10.300 cell/mm^3^, Hb of 12.9 g/dL, and platelets of 35.7000/mm^3^. Laboratory investigations were performed to exclude diabetes mellitus and prolactin deficiency. The levels of antibodies in the serum are shown in Table 1. Nephelometry was used to determine the total levels of IgG subclasses. The immunologic profile abnormalities including low levels of serum IgG4 and decreased ratio of CD4 to CD8 are shown in Table 2. Considering the IgG4 deficiency, we started IVIG 200 mg/kg monthly; the patient is now pregnant with gestational age of 32 weeks.

## Discussion

This study presents two women with low levels of IgG4 and primary RM. After treatment with IVIG, they had successful pregnancies. To date, there have been very few reports of IgG4 deficiency in women with RM^(12)^. A normal pregnancy is associated with increased levels of interleukin (IL)-10 and IL-4 related to Th2 type, whereas some miscarriages are associated with high levels of interferon-gamma and IL-12 related to Th1 type. In normal pregnancy, secretion of IL-4 causes the plasma cells to switch to the production of IgG4, whereas in miscarriage, elevated levels of interferon-gamma inhibits the process of switch to IgG4^(4)^. Wilson et al.^(11)^ study showed that women with RM had significantly low levels of total IgG and IgG subclasses 1, 2, 3, and 4 compared with normal pregnant women. IgG4 is an antibody with unique biologic properties^(13)^. There is evidence for the beneficial effect of IgG4 in allergic disease by inhibiting mast cell degranulation and impairing anti-tumor immunity in malignant melanoma; however, the mechanism of IgG4 in normal pregnancy is not fully understood. IgG4 generally induces a tolerance mechanism in allergic immunotherapy; therefore, it may play an important role in maternal tolerance to the fetus and maintenance of pregnancy. A new clinical study by Piccinni et al.^(14)^ suggests that the local production of leukemia inhibitory factor (LIF) is necessary for embryo implantation and it can be up-regulated by IL-4 and progesterone. We think IgG4 may contribute to LIF production; low level of IgG4 followed by miscarriage. The level of IgG4 in our two cases was very low. It is notable that as many as 10-15% of normal people have IgG4 concentrations below the limit of detection^(15)^. A prominent feature in the CD markers of our patients was the decreased the ratio of CD3CD4 T cells to CD3CD8 T cells. A normal CD4:CD8 ratio in pregnant adults is not 2:0; Ghafourian et al.^(16)^ investigated different types of T-cell subsets in 25 women with RM. Compared with normal, the proportion of CD3CD8 T cells was significantly higher in women with RM. IVIG was administered for the treatment of our cases with successful results, similar to Abdou et al.^(17)^ study. The mechanism of IVIG in the treatment of women with RM is the modulation of immune cells. A recent study found that IVIG increased the regulatory T cells and diminished Th 17 responses in RM^(18)^. Although we did not measure post-treatment IgG4 levels in our patients, it could explain the possible beneficial effect of IVIG on the increased levels of IgG4 in women with RM and deficiency of IgG4. We recommend IgG4 subclass determination in the initial evaluation of women with RM. We need a deeper understanding of the role of IgG4 in pregnancy; it is expected that these results will shed further light on the feasibility of IVIG for women with RM.

## Figures and Tables

**Table 1:**
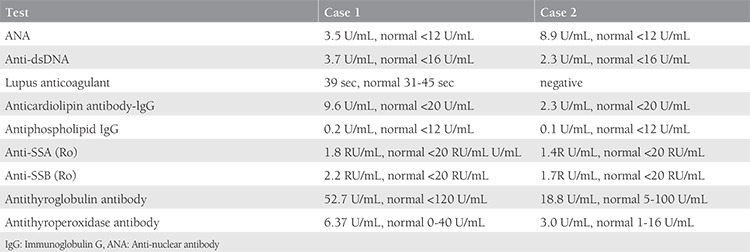
Different antibody identification in the patients with recurrent miscarriage

**Table 2:**
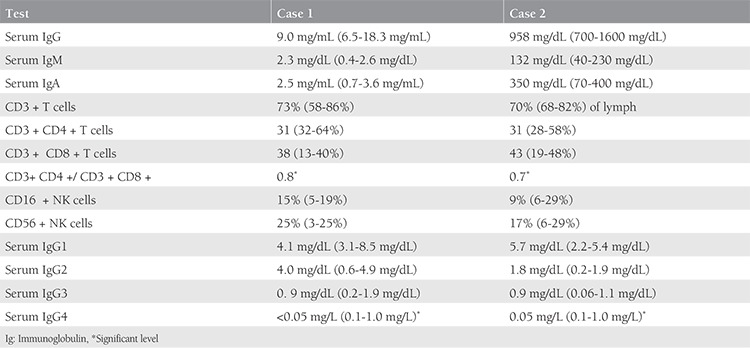
Immunologic investigations in the patients with recurrent miscarriage
